# Sulphur in Melancholia

**Published:** 1888-07

**Authors:** J. M. Dutton

**Affiliations:** Boston, Mass.


### SULPHUR IN MELANCHOLIA.

There are several points in the following history which make it seem worthy of record to a young physician: the length and gravity of the disease; the contrast between mixed and pure Homœopathy as curative forces; the power of the single dose and the long continuance of its action. A. E. G., æt. twenty-six, consulted me in the spring of 1886. Invariably cheerful and light of heart as a child, she had began seven years previously to suffer occasionally with mental depression. This tendency was much increased by the protracted illness and painful death of a favorite sister two years before. Since that time gloom had gradually taken possession of her life till death seemed the only refuge from her misery. So dominated was she by this idea of suicide that her family were gradually and painfully deciding that she could not be properly guarded outside of an asylum. The family history gave no evidence of insanity, but the father suffered from a chronic conjunctivitis and the children all had soft, unwholesome flesh, with pasty complexions. My note-book gives the following symptoms: Great dejection with thoughts of suicide. Feels inferior to everybody. Can decide nothing. This was so marked that she would not get down to breakfast until eleven o'clock, if left to herself, because she had nothing suitable to wear. When visiting she detained the company from table while she made an agonizing but futile effort to decide whether boots or slippers were to be worn. Listless, unable to work. Sometimes can understand nothing she reads.

Great disgust, amounting to nausea, at the odors of her own body. Chronic catarrh, with a bland yellow discharge. A lump comes in her throat; she must swallow continually; worse in a warm room. Constipation with frequent ineffectual desire for stool with passage of fœted flatus which disgusts her much. Amenorrhœa of five months' standing. There had been no effort of nature to establish the flow. A sense of weight in the pelvic region when walking. A muddy complexion with occasional ache on face.

The patient had been under the care of a prominent physician of this city during several months. He had à far wider medical experience than I could bring to the case. He was, however, an advocate of modern homœopathic liberalism as taught by Dr. Hughes. The treatment had been black pills, put up by the neighboring druggist, presumably aperient, since they were to be followed by enemata, Hunyada water, then last, and undoubtedly least in the company they were compelled to keep, homœopathic globules. This course of treatment, apparently so dynamic, had proved itself utterly powerless to alter one diseased condition. Examining the symptoms with the aid of Lippe's *Repertory*, two hours' work showed that Sulphur was found under all but two, while "disgust amounting to nausea at the effluvia of one's own body," a symptom much dwelt on by the patient, was found under Sulphur alone. Sulphur$^{cm}$, Swan, was given, repeating twice. The third day after the first dose there was a painless natural movement of the bowels, the first one without the aid of pills or enemata since six weeks. Here constipation as a feature of the case disappeared, a daily movement of the bowels being the habit up to this time. After a fortnight there was an attempt at menstruation; at the end of six weeks the catamenia appeared, being normal in amount and painless.

Almost immediately after the exhibition of Sulphur the mental symptoms began to improve, the change being gradual but continuous. There was never a day during the first three months when one could not say the patient is mentally slightly better than yesterday. At the end of six months a curious revulsive action was observed. Perhaps we should more accurately call it a proving of the Sulphur symptom, "ordinary objects cause extraordinary delight." She who for years had taken scant pleasure in her friends, and had latterly absolutely fled from the hateful sight of men, now found every one gifted with the most beautiful characters. To some of us drudging mortals, a little world weary and strangers to the rejuvenating action of Sulphur, the patient's ecstatic tales about what seemed to us the ordinary doings of ordinary mortals were a little fatiguing. There were during the first three months digestive disturbances which suggested Nux vomica and again Lycopodium. In view, however, of the continuous improvement in the mental sphere no one who had read the *Organon* could have ventured to interfere. The patient received no further medication. To-day all functions are normal and her life is a pleasure to herself and her friends.

In reviewing this report, one fresh from the methods of the old school can hardly avoid drawing the following conclusions: Here was a disease, possessing none of the characteristics of self-limitation, whose course seems to have been altered by means of a medicinal agent. The old school armamentarium possesses nothing capable of producing a like effect. The physician showed only ordinary ability in studying the case. The "prentice hand" appears plainly in taking the symptoms, no clear apprehension of their modality having at that time been reached. The only trait shown was fidelity to the teachings of Hahnemann, and only through such faithfulness can the superiority of the methods of the master be shown.

J. M. Dutton, M. D.

Boston, Mass.

